# Non-Invasive Brain Stimulation: Augmenting the Training and Performance Potential in Esports Players

**DOI:** 10.3390/brainsci10070454

**Published:** 2020-07-15

**Authors:** Wei Zhuang, Keyi Yin, Yahua Zi, Yu Liu

**Affiliations:** School of kinesiology, Shanghai University of Sport, Shanghai 200438, China; zhuangweisus@outlook.com (W.Z.); yky0504@outlook.com (K.Y.); ziyahua@hotmail.com (Y.Z.)

**Keywords:** competitive computer gaming, electronic sports, performance, skill acquisition, sports

## Abstract

During the last two decades, esports, a highly competitive sporting activity, has gained increasing popularity. Both performance and competition in esports require players to have fine motor skills and physical and cognitive abilities in controlling and manipulating digital activities in a virtual environment. While strategies for building and improving skills and abilities are crucial for successful gaming performance, few effective training approaches exist in the fast-growing area of competitive esports. In this paper, we describe a non-invasive brain stimulation (NIBS) approach and highlight the relevance and potential areas for research while being cognizant of various technical, safety, and ethical issues related to NIBS when applied to esports.

## 1. Introduction

Esports, or digital sports, is an emerging form of sport facilitated by electronic systems. Esports is now broadly viewed as a competitive sporting activity [1,2,3] and in recent years has enjoyed fast-growing popularity, both professionally and among amateur players [4,5]. Despite the highly competitive nature of the activity, which is both physically intense and mentally demanding [3,6], relatively little is known regarding effective esports training strategies designed to develop fine motor skills and optimize performance [7]. In this paper, we present a non-invasive brain stimulation (NIBS) approach [8] for esports training that has gained increasing attention in the non-digital (traditional) sports literature [9,10,11]. We highlight the relevance of NIBS to esports in facilitating skill acquisition and improving motor and cognitive performance. We also describe safety issues and caveats associated with the use of NIBS-based techniques when applied to performance enhancement among esports players. 

## 2. Essential Motor and Cognitive Skills in Esports 

In esports, groups of players compete against others in competitive video games on personal computers or gaming consoles. Therefore, training and competing in esports primarily involves the use of control devices (e.g., keyboards, mice, or console controllers). Playing esports requires efficient manual dexterity, good hand–eye coordination, highly focused attention, fast reaction times, and rapid decision making [3,6] in a virtual and electronic environment [1]. Esports players need to have strong physical, cognitive, and mental skills to endure long hours of daily training and must learn to maneuver through fast-changing, unpredictable virtual environments to succeed in highly intense and fiercely competitive tournaments. Many important esports skills also involve fine motor skills that combine precise and accurately controlled movements with cognitive decision-making skills that may take months or even years of practice to master [3]. Thus, the characteristics of esports require that a holistic training approach, such as exergaming [7], be adopted. 

## 3. NIBS as a Neuromodulatory Technique of Brain Function

In what follows, we describe the application of non-invasive brain stimulation (NIBS), a neuromodulation technique for non-invasively stimulating the brain and central nervous system, for augmenting the performance of esports players, and for developing the skills they specifically need at the physical, cognitive, and mental levels.

NIBS mainly involves the use of electrical currents or magnetic fields to stimulate targeted regions of the brain. The rapid development of NIBS techniques in the past two decades has made a large contribution to neuroscience [8,12], and these techniques have become more accessible in clinical and non-clinical settings, such as sports [9,10,11,13,14]. Recently, cortical stimulation approaches, including transcranial current stimulation (tCS), which includes transcranial direct current stimulation (tDCS) and transcranial alternating current stimulation (tACS), as well as transcranial magnetic stimulation (TMS), have all become well-established.

In tDCS, a device with two small electrodes (a positive “anode” and negative “cathode”) is placed on the head in order to deliver a constant low level of electric current (1 to 2 mA) that results in the alteration of neuronal excitability. tACS, instead of applying a direct electrical current to the brain, oscillates a sinusoidal current at a chosen frequency that interacts with the brain’s natural cortical oscillations. In TMS, an electromagnet is placed on the scalp. After the apparatus has been turned on, the coils of the electromagnet change polarity, producing short magnetic pulses that result in activation of axons in the brain, thus leading to fire action potentials. TMS can be applied in single pulses, pairs of pulses, or repeated trains of pulses (rTMS) [15]. Relative to TMS and tACS, tDCS is currently the most frequently used technique in the field of sport and exercise science [9]. Table 1 summarizes the major methodological characteristics, stimulation protocols, and potential risks associated with tCS and TMS techniques.

Studies have shown therapeutic benefits from the application of NIBS-based techniques in clinical populations [16,17,18]. In the area of sports activities, several comprehensive reviews are available [9,10,11,13,14] that summarize the benefits of applying tDCS/tACS and TMS techniques for facilitating motor learning and motor skills. Indeed, there has been an increasing interest in exploring the potential of using NIBS-based techniques in multiple domains, including physical capability and athletic performance [13], as well as muscular strength, endurance, and fatigue [9,14]. Additional evidence also suggests the effectiveness of NIBS in improving working memory [19,20], decision making [21], attention [22,23], multi-tasking [24], reaction time [25], and motor learning and skill acquisition [26,27,28]. These performance-related outcomes are highly relevant to the training and performance characteristics of esports [6,7]. 

**Table 1:** Summary of methodological characteristics, stimulation parameters, and potential risks associated with transcranial current stimulation (tCS) and transcranial magnetic stimulation (TMS) techniques.

NIBS Methods	NIBS Techniques	Polarity for tES and Pulse Mode for TMS	Variable Parameter	Current for tES(mA) and Pulses Per Session for TMS	Duration of Each Session (min)	Risks	References
tES methods	tDCS	Polar	Anodic stimulation: excitatory effect Cathodic stimulation: inhibitory effect	0.5–2	5–30	Mild burning/Itching sensation/Mild headaches/Fatigue	Nitsche MA., and Paulus W. [29,30]; Dedoncker J., et al. [31]; Dissanayaka T., et al. [32]
	tACS	Alternating	Frequency (0.1–640 Hz)	0.5–2	5–30		
	tRNS	Alternating	Frequency	0.5–2	5–30		
rTMS methods	HF	Single pulse	≥10 Hz	3000	30	Headache/Scalp discomfort/Tingling, spasms or twitching of facial muscles/Lightheadedness	Rosa MA., and Lisanby SH., [33]; Rossi S., et al. [34]; Huang YZ., et al. [35]
	LF	Single pulse	≤1 Hz	1200	20		
	iTBS	Pulses per burst 3 (at 50 Hz)	5 Hz	600–900	4–7		
	cTBS	Pulses per burst 3 (at 50 Hz)	5 Hz	600–900	2–3		

NIBS = non-invasive brain stimulation; tES = transcranial electric stimulation; tDCS = transcranial direct current stimulation; tACS = transcranial alternating current stimulation; tRNS = transcranial random noise stimulation; TMS = transcranial magnetic stimulation; TBS = continuous theta-burst stimulation; HF = high frequency; iTBS = intermittent theta-burst stimulation; LF = low frequency; rTMS = repetitive transcranial magnetic stimulation.

## 4. Potential Benefits and Areas of NIBS Applications in Esports

**Figure 1:**
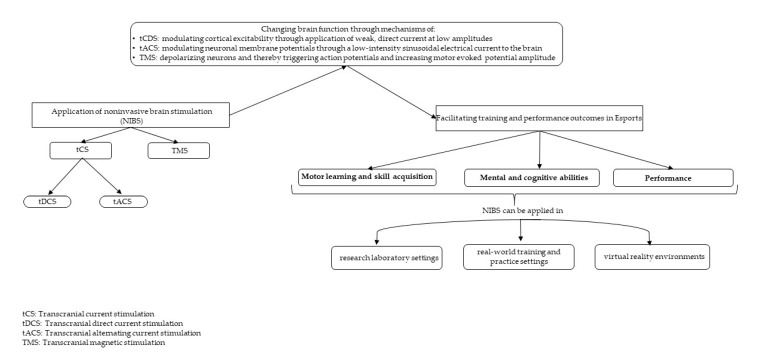
A framework for potential research on non-invasive brain stimulation in esports.

Just as in traditional sports, playing and competing in esports gaming requires fine motor skills, mental agility, and cognitive ability [3,6]. Therefore, benefits of NIBS observed in the sports literature may have direct implications for esports and can thus serve as a scientific premise for exploring the practical utility of NIBS-based techniques in improving skill acquisition and performance among esports players [9]. In what follows, we highlight a few areas of research relevant to esports (see Figure 1 for a schematic representation of our proposed framework for potential research), and Table 2 briefly summarizes the studies for the potential benefits of NIBS applications in esports.

***Improving manual dexterity***. Esports players can reach up to 400 keystrokes per minute, suggesting a highly demanding activity that requires a high level of dexterity [7,36]. NIBS techniques such as tDCS and high-definition tDCS, when applied to premotor and primary motor cortices, have been shown to improve motor performance of unimanual [37] and bimanual dexterity in healthy adults [38]. These outcomes suggest that tDCS may be applied as a training protocol aimed at improving manual dexterity. 

***Improving physical exertion***. Esports performance is both physically and mentally demanding and requires great physical exertion, with increased heart rates up to 160 to 180 beats per minute, especially during competition [36]. Research using anodal tDCS over the left temporal cortex (an area that is associated with autonomic nervous system (ANS) control) has been found to modulate activity in the ANS and alter rating of perceived exertion and improve exercise performance (i.e., peak power output) by 4% [39]. Similarly, Kamali et al. showed that, compared to those in a sham condition, bodybuilders who received tDCS in the primary motor cortex and left temporal cortex experienced significant reductions in physical exertion and heart rate and improvements in strength and endurance during performance of knee extension exercise [40]. Another study showed that, compared to sham stimulation, the application of anodal tDCS during the performance of a fatiguing activity significantly increased time to task failure [41]. These findings indicate that physical exertion and fatigue, which fit the competitive profile of esports training, can be modulated through proper tDCS and that tDCS has the potential to augment the capability of performing and competing under the intense and challenging conditions of esports. 

***Effects on reaction time***. Perceptual reaction times are crucial in esports. Therefore, understanding whether NIBS can positively impact performance on reaction times is of practical importance. The evidence, however, is inconclusive. In one study [25], healthy adults received real (with 1 mA) or sham tDCS over their dorsolateral prefrontal cortices during two 30-min mathematics training sessions involving body movements. To examine the impact of training, an active control group received tDCS during a non-mathematical task. Results showed that 2 months after the training, participants who received real tDCS performed significantly better in game response times (20% faster) and accuracy than the sham group, indicating that 2 days of 30-min training with tDCS could have long-lasting impact on neuroplasticity. However, in a recent study, Seidel and Ragert [42] showed that, compared to a sham condition, an application of a 20-min anodal tDCS over the primary motor cortex (leg area) resulted in no tDCS-induced change on reaction time and tapping performance tasks of the lower extremity for both athletes and non-athletes. Results from this study suggest that neither athletes nor non-athletes benefit from a brief period of tDCS application in speed-related motor tasks.

***Improving motor learning and skill acquisition***. Techniques such as tDCS and tACS have been shown to improve motor learning and skill acquisition, which make them applicable in esports training. One study showed that anodal tDCS of the primary motor cortex increased performance of a serial reaction-time task, suggesting involvement of the primary motor cortex in skill acquisition and early consolidation phase of implicit motor learning [43]. Another study showed that, compared to a sham condition, greater total skill acquisition in learning a novel and challenging motor skill task occurred when anodal tDCS was applied [27]. In studies involving tACS, research has shown that when tACS is applied over the left primary motor cortex within the alpha- and beta-frequency bands, the stimulation significantly improves sequence learning, as indexed by a serial reaction-time task, and promotes quicker skill acquisition [44]. A review by Luber and Lisanby [45] provided some evidence that TMS-modulated cortical networks produce cognitive performance enhancements in a variety of tasks involving perceptual, motor, and executive processes in healthy individuals.

***Improving endurance***. Esports players often undergo long training hours daily [46,47] and thus spend excessive time in a sitting position [48], which argues for the importance of having muscular endurance for efficiently practicing esports skills. NIBS may help players to increase endurance for sustaining strenuous daily training. One study showed that tDCS, with the anode over both motor cortices and using a bilateral extracephalic reference, improved endurance performance among healthy adults during a cycling time-to-task-failure test [48]. Specifically, the researchers assessed neuromuscular performance, both before and after tDCS, by measuring time to task failure among participants engaged in cycling sessions. The results of the study showed that placing the anodes over both motor cortices augmented the cyclists’ endurance; thus, those who received anodal stimulation biked longer before quitting than did those under the cathodal and sham conditions.

Several review articles [9,10,11,13,14] have identified other areas where NIBS may be applicable and beneficial to esports players. These areas include muscular strength, motor coordination, and motor sequence learning. 

**Table 2:** A summary of studies for the potential benefits and areas of non-invasive brain stimulation (NIBS) applications in esports.

Potential Benefits and Areas	Relevant Skills and Abilities in Esports	NIBS Techniques	Main Effects	Study
Performance	Finger speed and dexterity	tDCS/HD-tDCS	Improving motor performance of unimanual and bimanual dexterity	Pavlova E., et al. [37]; Pixa NH., et al. [38]
	Hand–eye coordination	tDCS	Enhancing visuo-motor learning and visuomotor coordination	Antal A., et al. [49,50]; Kwon YH., et al. [51]
	Reaction time	tDCS/tACS	Improving performance in gaming response times; Shortening reaction time to solve complex logic problem;	Looi CY., et al. [25]; Santarnecchi E., et al. [52];
	Movement precision and muscle control	tDCS	Enhancing precise hand movement and proprioception	Matsuo A., et al. [53]; Beck E., et al. [54]
	Strength and power	tDCS	Improving performance-related capacities of athletes	Okano AH., et al. [39]; Kamail A., et al. [40]
	Endurance	tDCS	Extending time to task failure; Improving endurance performance	Williams PS., et al. [41]; Angius L., et al. [48]
Mental & cognitive abilities	Decision making	tDCS	Producing a reliable speeding of response times during decision-making; Enhancing advantageous decision-making	Filmer HL., et al. [55]; Julien O., et al. [21]
Mental & cognitive abilities	Working memory	HD-tDCS/tACS/rTMS	Increasing learning rates of performance metrics; Increasing working memory storage capacity score; Improving n-back task performance	Ke Y., et al. [56]; Jausovec N., et al. [57] Esslinger C., et al. [58]
	Multi-tasking	tDCS	Enhancing performance for multi-tasking paradigm and visual search tasks; Improving information processing capabilities during a multi-tasking environment	Filmer HL., et al. [59] Nelson J., et al. [60]
	Attention control	tDCS/tACS	Improving executive attention; Improving performance of a visual search attention task; Decreasing reaction time in a continuous performance test	Miler JA., et al. [61]; Mauri P., et al. [62] Müller, NG., et al. [63]
Motor Learning & skill acquisition	Motor learning speed	tACS/TMS	Stabilizing the newly learned motor task; Enhancing motor skill acquisition	Pollob B., et al. [44]; Butts RJ., et al. [64]
	Movement coordination	tDCS	Improving motor adaptation in the upper limb; Fasting intentional switches between coordination patterns	Weightman M., et al. [65]; Carter MJ., et al. [66]
	Acquisition of complex motor skills	tDCS	Increasing greater total skill acquisition; improving implicit motor learning	Reis J., et al. [27]; Nitsche MA., et al. [43]

## 5. Safety and Risk Factors Related to NIBS

Although there are currently no safety guidelines with respect to tES, both TMS and tDCS techniques have generally been shown to be safe for use in human subjects [9,34,67]. Although tDCS is not currently approved by the U.S. Food and Drug Administration for clinical use, TMS is as an approved treatment modality for depression [68]. However, there are some safety and risk factors that researchers, practitioners, and clinicians alike should be aware of. These include (a) both acute and chronic NIBS, (b) the potential long-term adverse effects of prolonged stimulation or repetitive application of NIBS, and (c) individual differences (e.g., sex) in response to NIBS [69,70]. These issues are magnified in light of the fact that most players, including professional players, are children and adolescents [71,72], which puts them at greater risk.

## 6. Caveats

Although most studies show positive effects on motor skills from the application of NIBS techniques, the underlying mechanisms through which each of these techniques influences the outcomes of interest remain largely unexplored and can be highly complex [70]. It is, however, commonly postulated that application of a stimulation (e.g., anodal tDCS) to a targeted brain area induces brain (cortical neuron) excitability, which elicits action potentials that can subsequently increase motor output and therefore improve performance ability [9,73]. Other researchers suggest that the excitability resulting from targeted stimulation may reduce the need for generating effortful output required for muscle recruitment. This, in turn, may result in a low perception of exertion for a given force or power output, which may serve as a mechanism for improved performance [14]. Simulation techniques such as cathodal tDCS decrease cortical excitability (i.e., inhibition) [74]. 

There remain many methodological issues that must be addressed before the evidence is conclusive on the effectiveness of NIBS in improving motor and cognitive performance in esports [9]. For example, in addition to significant variability of NIBS-induced effects [75], optimized experimental protocols such as stimulation duration, electrode montage, and stimulation amplitude for applying tDCS techniques remain to be determined. Furthermore, because many studies have used an experimental design, the extent to which NIBS’s enhancement effects can be meaningfully generalized to actual sport competitions remains unknown [76,77]. 

Drawing exact boundaries for using NIBS techniques for improving performance-related training outcomes and enhancing performance immediately prior to competition for the sole purpose of winning the event (and perhaps winning prize money) are challenging. The application of NIBS techniques in real-world settings is not currently regulated by any group of technical experts—governmental, academic, or otherwise. However, there is consensus in the scientific community that NIBS should not be intentionally used as a neurodoping or ergogenic aid in seeking a “marginal gain” or “elusive edge” in performance during competition [11,78,79]. With the increasing use of NIBS in sports and esports, ethics and regulatory guidelines will need to be established in order to avoid misuse of these neuroenhancement techniques as a tool for supercharging performance in sports. 

Finally, we are fully cognizant of the many health issues that participating in esports [6,80] can raise, including gambling disorders, overuse injuries, and doping behaviors. These issues constitute yet another avenue for research in which the use of neuromodulation can address the negative health consequences of esports. 

## 7. Conclusions

The use of NIBS as a neuromodulation technique has received increasing research attention. The application of NIBS in the fields of sports and physical exercise has led to mounting evidence that suggests both health and performance benefits. Though this research is in its infancy, this evidence has provided the scientific premise and impetus for exploring the potential of NIBS-based techniques in helping improve learning and performance of motor and cognitive skills in esports. Within this context, we highlight the relevance of NIBS to esports and the potential areas in which NIBS can be integrated into esports training (Figure 1). At the same time, we remain cognizant of the various technical, ethical, and regulatory aspects of NIBS when applied to esports.
